# A Fluorescence-Based Assay for Measuring the Redox Potential of 5-Lipoxygenase Inhibitors

**DOI:** 10.1371/journal.pone.0087708

**Published:** 2014-02-03

**Authors:** Sangchul Lee, Youngsam Park, Junghwan Kim, Sung-Jun Han

**Affiliations:** Institut Pasteur Korea, Seongnam-si, Gyeonggi-do, Korea; Fundação Oswaldo Cruz, Brazil

## Abstract

The activities and side effects of 5-lipoxygenase (5-LO) inhibitors can be predicted by identifying their redox mechanisms. In this study, we developed a fluorescence-based method to measure the redox potential of 5-LO inhibitors and compared it to the conventional, absorbance-based method. After the pseudo-peroxidase reaction, the amount of remaining lipid peroxide was quantified using the H2DCFDA (2′,7′-dichlorodihydrofluorescein diacetate) fluorescence dye. Our method showed large signal windows and provided comparable redox potential values. Importantly, the redox mechanisms of known inhibitors were accurately measured with the fluorescence assay, whereas the conventional, absorbance-based method showed contradictory results. Our findings suggest that our developed method is a better alternative for classifying the redox potential of 5-LO inhibitors, and the fluorescence assay can be effectively used to study the mechanisms of action that are related to redox cycling.

## Introduction

Leukotrienes (LTs) play important roles in immune responses. Leukotriene B4 (LTB4) recruits neutrophils to damaged tissue and induces the production of inflammatory cytokines. Cysteinyl LTs are involved in endothelial cell adherence and chemokine production [1]. They also increase muscle contractions to reduce airflow in asthma, and anti-LTs are used to treat asthma [2]. Leukotriene A4 (LTA4) is produced by two consecutive steps of dioxygenation from arachidonic acid by 5-lipoxygenase (5-LO). LTA4 is then converted to LTB4 by LTA4 hydrolase, or to cysteinyl LTs by LTC4 synthase and other related enzymes [1]. Because 5-LO plays an essential role in the production of various LTs, its inhibition is expected to be the most effective in treating diseases caused by overproduction of LTs, such as asthma, arthritis, pulmonary hypertension, atherosclerosis, osteoporosis, and prostate cancer [3], [4].

Many 5-LO inhibitors have been developed to treat inflammation-related diseases. Depending on their actions at the ferric iron, which is at the center of the 5-LO active site, they are conventionally classified into three categories: redox inhibitor, iron ligand inhibitor, and non-redox inhibitor [5]. During the process of enzyme activation, lipid peroxide converts inactive 5-LO with ferrous iron into active 5-LO with ferric iron. Redox inhibitors reduce ferric iron to inactive ferrous iron. Iron ligand inhibitors have binding affinity to the ferric iron and block the binding ability of substrates without changing the iron state. Non-redox inhibitors compete with substrates for binding to 5-LO [6]. Estimating the redox characteristics of an inhibitor is important in understanding its actions in various diseases. Redox-active inhibitors are usually lipophilic-reducing agents, and poor selectivity can cause side effects, such as methemoglobinemia, through actions on other redox systems that utilize ferric irons in the body [7]. On the other hand, non-redox 5-LO inhibitors are highly potent in the low nanomolar ranges of IC_50_; however, they show impaired potency in a condition with elevated peroxide levels [8]. Thus, elucidating the mechanisms of each class of inhibitors requires additional experiments. Substrate specificity is more important for redox inhibitors, whereas pathophysiologically relevant tests are required for non-redox inhibitors.

**Figure 1:**
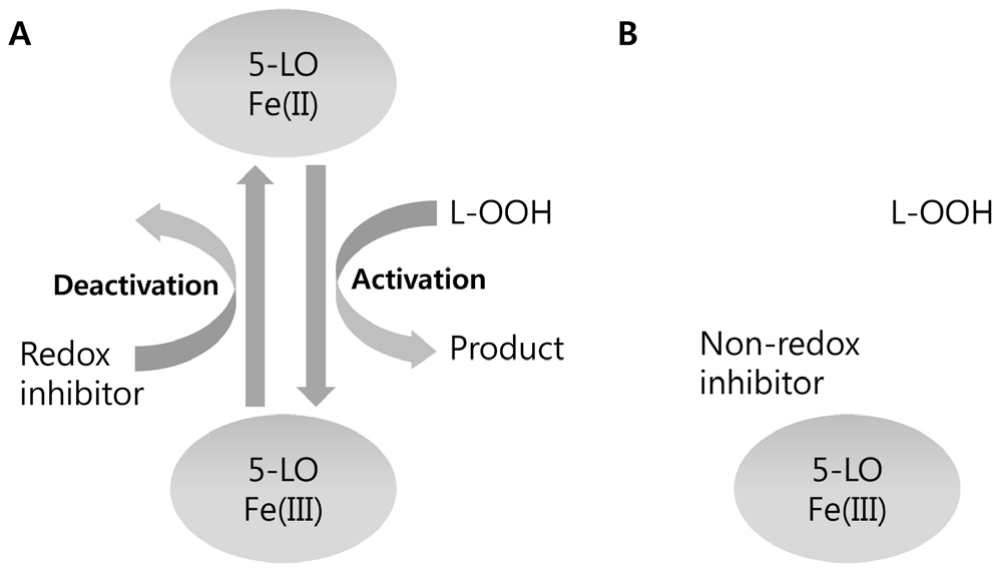
Measurement of lipid peroxide (L-OOH) as the redox determinant of 5-LO inhibitors. (A) In the presence of a redox inhibitor, the active 5-LO is continuously deactivated to its ferrous form. It can then be re-activated by consumption of L-OOH. (B) Non-redox inhibitors are devoid of redox activity. Active 5-LO binds to the inhibitor, but iron stays in its active, ferric form, and L-OOH is not involved in the reaction. Thus, the amount of L-OOH does not change. The reduced amount of L-OOH signifies the redox activity of the inhibitor.

Measuring the pseudo-peroxidase activity of 5-LO in the presence of its inhibitor is a way to determine the redox activity [Figure 1] [9]. An inhibitor that has redox activity converts the ferric enzyme into a ferrous state. Subsequently, lipid peroxide is consumed to bring the ferrous enzyme back to the ferric state. The reduction in lipid peroxide concentration is an indicator of redox activity, and it can be measured by the decrease in absorbance of the lipid peroxide itself. This method has been qualitatively and quantitatively used in several studies [10], [11]. However, obtaining comparable quantitative values among redox inhibitors is difficult, due to the small changes in absorbance and the rapid velocity by which pseudo-peroxidase activity can increase at the beginning of the reaction.

In this study, we developed a fluorescence-based 5-LO redox assay that measures the amount of peroxide by using a sensitive fluorescence dye. Upon cleavage of the acetate groups by intracellular esterases and oxidation by peroxide, the nonfluorescent H2DCFDA is converted to the highly fluorescent 2′,7′-dichlorofluorescein, and the resulting fluorescence values provides a large signal window. Dose-response curves can be generated by this method, thus allowing the effective concentration of inhibitor (EC_50_) needed to yield redox potential to be calculated. Several known redox and non-redox inhibitors were tested using this method. While the absorbance-based method yielded many contradictory mechanisms for the tested inhibitors, the fluorescence-based method provided accurate, corresponding mechanisms. Our results suggest that the fluorescence-based assay may be a good tool for assessing the mechanisms of action in relation to redox cycling.

## Materials and Methods

### Materials

H2DCFDA (2′,7′-dichlorodihydrofluorescein diacetate) was purchased from Life Technologies (Carlsbad, CA, USA). Zileuton (N-[1-benzo(b)thien-2-ylethyl]-N-hydroxy-urea, CAS 111406-87-2) was purchased from Sigma-Aldrich (St. Louis, MO, USA) and NDGA (4,4′-(2,3-dimethyl-1,4-butanediyl)bis-1,2-benzenediol, CAS 500-38-9) was purchased from Cayman Chemical (Ann Arbor, MI, USA).

Human recombinant 5-LO lysate, 13(S)-HpODE (13S-hydroperoxy-9Z,11E-octadecadienoic acid, CAS 33964-75-9), YS121 (2-[[4-chloro-6-[(2,3-dimethylphenyl)amino]-2-pyrimidinyl]thio]-octanoic acid, CAS 916482-17-2), caffeic acid (3,4-dihydroxy cinnamic acid, CAS 331-39-5), CDC (cinnamyl 3,4-dihydroxy-alpha-cyanocinnamate, CAS 132465-11-3), CAY10606 (2-[(3-chlorophenyl)methyl]-5-hydroxy-1H-benz[g]indole-3-carboxylic acid, ethyl ester, CAS 1159576-98-3), and CAY10649 ((Z)-2-(4-chlorophenyl)-5-(4-methoxybenzylidene)thiazol-4(5H)-one, CAS 1272519-89-7) were from Cayman Chemical. DMSO (dimethyl sulfoxide) was purchased from Sigma-Aldrich (St. Louis, MO, USA).

PF4191834 was kindly donated by Qurient, Inc. (Seongnam-si, South Korea).

Fluorescence and absorbance measurements were performed using the SpectraMax M5 microtiter plate reader (Molecular Devices, Sunnyvale, CA, USA).

### Selection of test compounds

We selected eight 5-LO inhibitors to be tested in the redox assays. NDGA is a strong antioxidant that inhibits 5-LO, 12-LO, and 15-LO through common redox mechanisms [12]. Zileuton is a unique and commercially available drug that targets 5-LO. It is categorized as an iron ligand inhibitor that also shows redox activity [9]. YS121 and CAY10649 are predicted to be non-redox inhibitors, based on their structures and functional moieties [13], [14]. Caffeic acid and its derivative, CDC, are redox inhibitors, according to a radical scavenging assay [15]. CAY10606 is also predicted to be redox-active, based on the fact that it is more potent than its derivative, which lacks redox moiety [16]. PF4191834 is a non-redox 5-LO inhibitor [17]. Overall, five of the eight selected compounds are known to have redox activity.

### Redox mechanism of inhibition

Lipid peroxide is consumed when 5-LO is activated to the ferric iron form. The enzyme remains activated during the dioxygenation reaction cycle. If a redox inhibitor is present, it reduces the catalytic iron into the ferrous form and inhibits the enzyme reaction. Another lipid peroxide must be consumed to re-activate 5-LO to the ferric form. As a result, reductions in the amount of lipid peroxide in the reaction mixture signify redox activity. A non-redox inhibitor does not change the iron state and, therefore, has no effect on the amount of lipid peroxide [Figure 1]. The absorbance-based method measures the amount of peroxide by its absorbance at 234 nm, and the fluorescence-based method measures it by analyzing its reaction with H2DCFDA.

### Redox fluorescence assay

H2DCF-DA (60 µM) was pre-cleaved by 5-LO crude lysate in the reaction buffer (50 mM Tris, pH 7.5, 2 mM ethylenediaminetetraacetic acid [EDTA], 2 mM CaCl_2_, and 10 µM adenosine triphosphate [ATP]) for more than 10 minutes before the main reaction, as previously reported [18]. In a 384-well plate, 10 µl of 450 mU/µl enzyme solution was distributed and mixed with 10 µl of inhibitor solution with various concentrations. 13(S)-HpODE (10 µM; 20 µl) was added to each well to start the reaction. After 3 minutes, pre-cleaved H2DCF-DA dye (20 µl) was added and incubated for more than 10 minutes. The fluorescence signal was measured using a fluorometer at excitation and emission wavelengths of 485 and 530 nm, respectively. All steps were carried out at room temperature.

### Redox absorbance assay

The redox absorbance assay was carried out as described by Zweifel, et al. [11]. Specifically, 10 µl of 1 mM inhibitor solution was mixed with 490 µl of 20 µM 13-HpODE solution. After 3 minutes of incubation at room temperature, the solution was transferred to a cuvette. The reaction started when 500 µl of the 20 unit/ml enzyme solution was added to the peroxide-inhibitor mixture in the cuvette. The absorbance of 13(S)-HpODE at 234 nm was measured immediately after mixing, using a SpectraMax M5 spectrophotometer. Data points were collected for 3 minutes. The reaction buffer consisted of 50 mM potassium phosphate buffer (pH 7.6), 0.1 mM EDTA, 100 µM ATP, and 0.3 mM CaCl_2_.

## Results

### Redox absorbance assay

The accuracy and efficacy of the redox absorbance assay were determined by testing the selected compounds. The absorbance of 13(S)-HpODE was measured at 234 nm, and the values were recorded every second for 3 minutes.

The starting absorbance values ranged from 1.908 to 2.305. These high values were due to contributions from the buffer, compounds, enzyme solution, and 13-HpODE. The changes in absorbance ranged from −0.042 (decrease) to 0.026 (increase). End-point measurements were not available, because the absorbance changes were less than the variations of the starting absorbance values. In theory, because the extinction coefficient of 13(S)-HpODE at 234 nm is 23,000 M^−1^ cm^−1^, 10 µM 13(S)-HpODE contributes a maximum of 0.23 in absorbance. The variations of the starting absorbance values are a result of the different absorptivity of the inhibitors. The results for all inhibitors were normalized by subtracting the starting absorbance values of the respective inhibitors.

**Figure 2:**
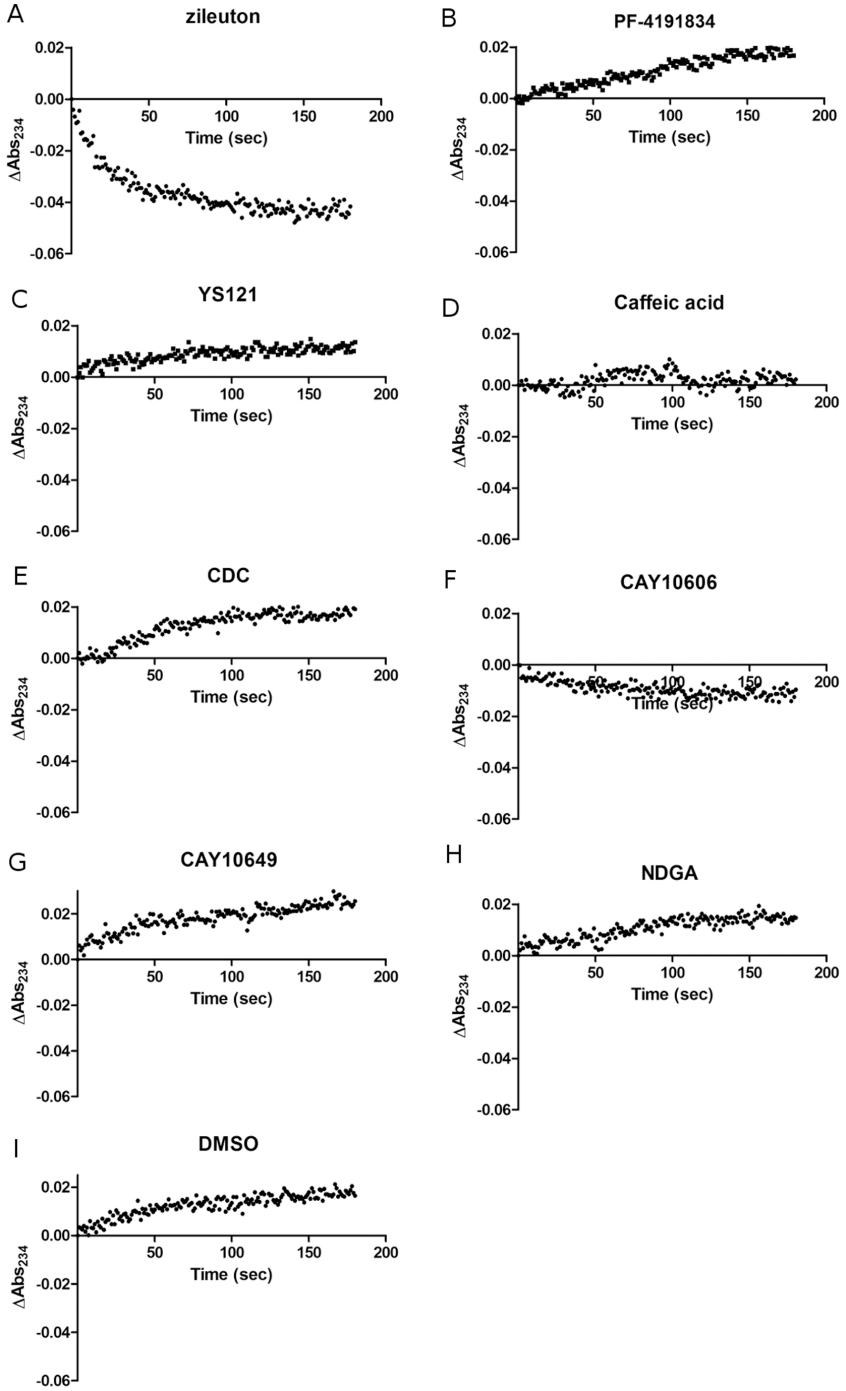
Kinetic curves of the redox absorbance assay. The kinetic curves of 13(S)-HpODE are depicted for 10 µM of (A) zileuton, (B) PF4191834, (C) YS121, (D) caffeic acid, (E) CDC, (F) CAY10606, (G) CAY10649, (H) NDGA, and (I) DMSO control. The amount of substrate was measured by absorbance at 234 nm, and measurements were recorded at every second for 3 minutes. The values were normalized by subtracting the absorbance at the start of the reactions. The average absorbance change of three independent tests were summarized in the Table 1. Redox inhibitors and non-redox inhibitors showed decreasing and increasing patterns, respectively.

**Figure 3:**
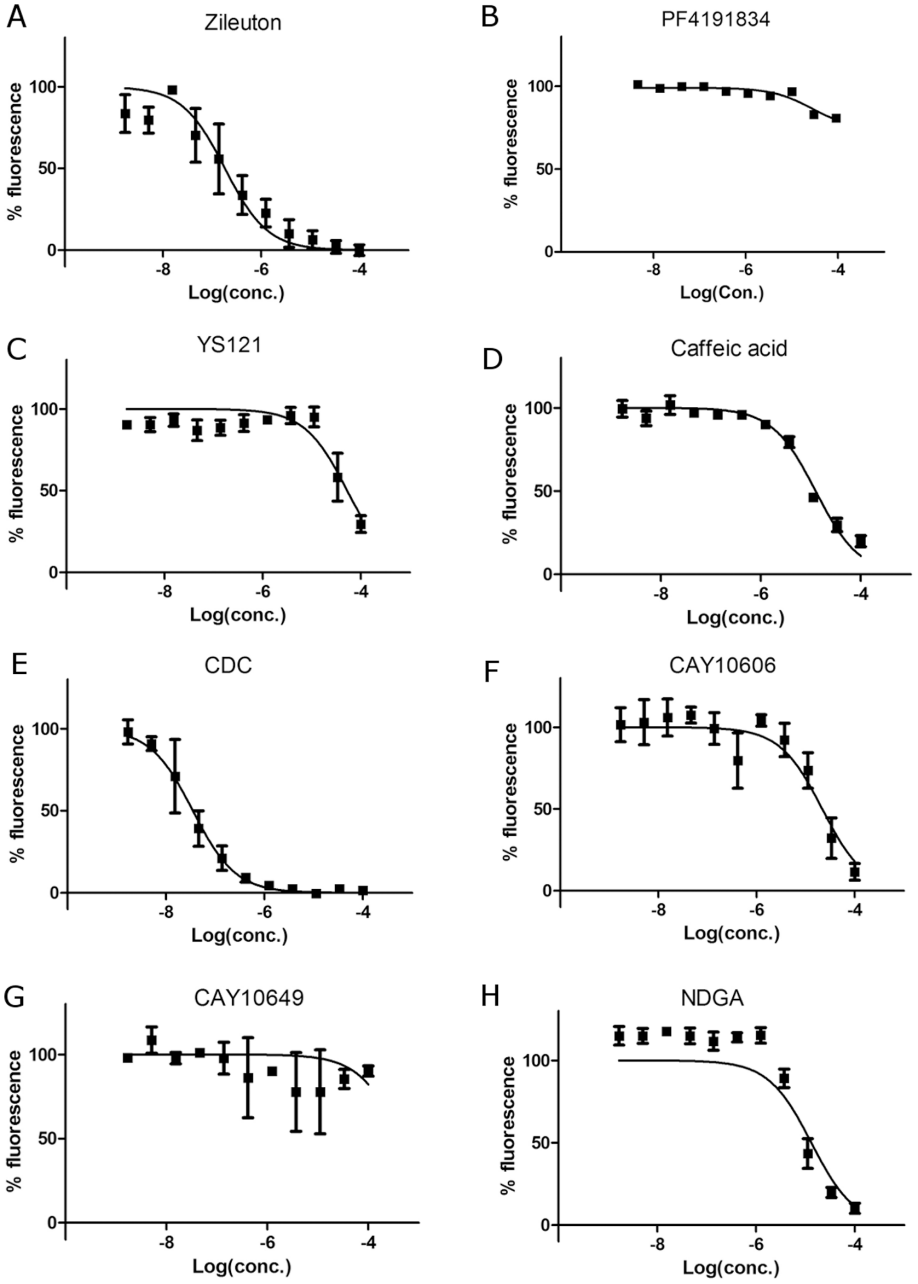
Dose-response curves of the redox fluorescence assay. The amount of remaining fluorescence represented the redox activity of the inhibitor. The concentration of inhibitors varied from 1.69 µM, and dose-response curves were generated for (A) zileuton, (B) PF4191834, (C) YS121, (D) caffeic acid, (E) CDC, (F) CAY10606, (G) CAY10649, and (H) NDGA. The tests were carried out in duplicate and each curve was fitted by three-parameter logistic regression, using the Prism software. Calculated EC_50_ values from three independent tests are averaged and listed in Table 1.

**Figure 4:**
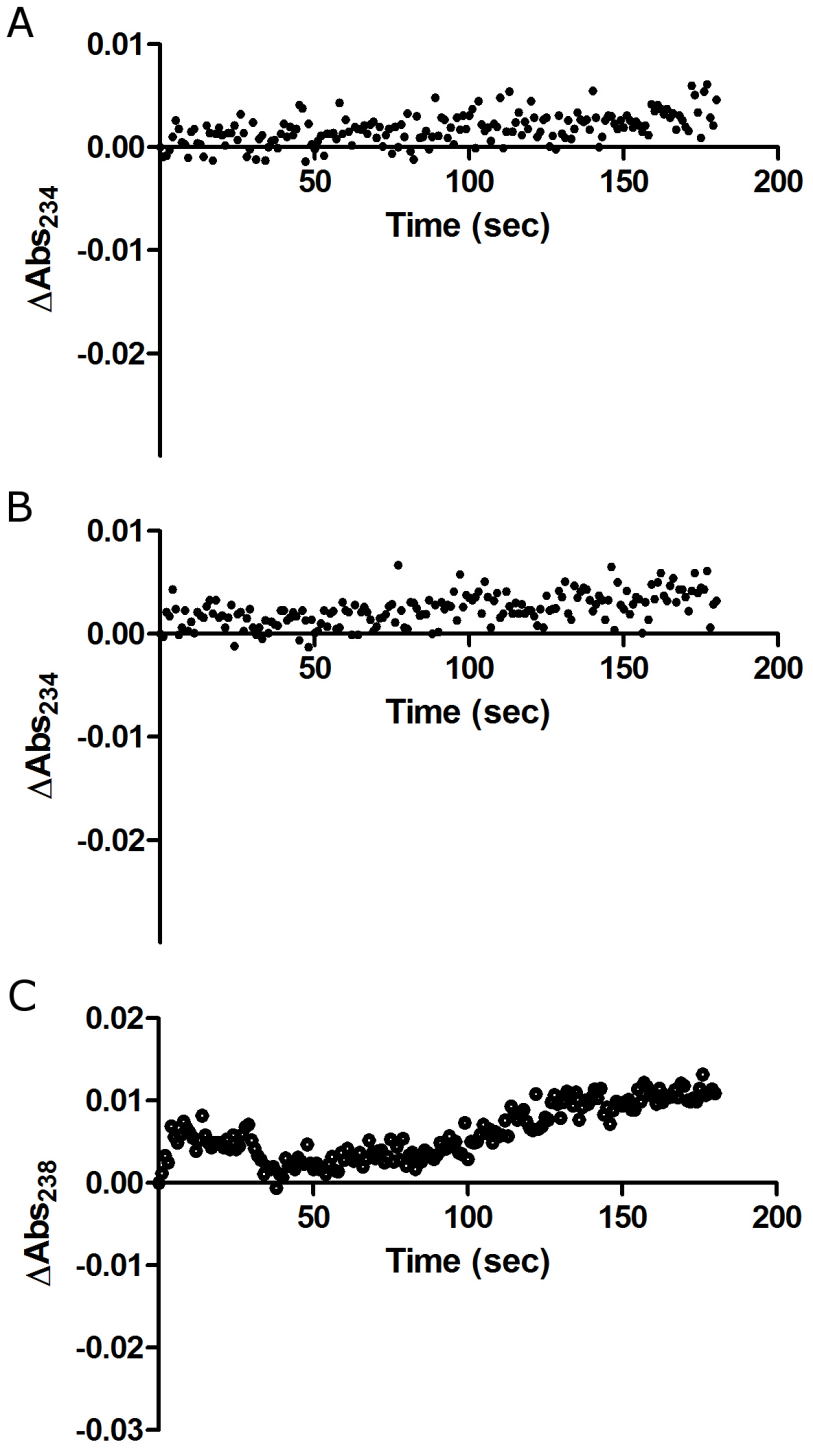
Redox absorbance assay with different conditions. To determine the source of discrepancies between the redox absorbance assay results and known mechanisms, the absorbance assay was carried out in different conditions. The patterns of redox absorbance kinetics in substrate-depleted conditions were tested with (A) zileuton and (B) DMSO at 80 minutes after the start of the reaction. The absorbance of zileuton increased slowly over time. (C) For CDC, which showed the strongest redox activity in the fluorescence assay, the absorbance assay was carried out in the buffer used in the fluorescence assay. However, redox activity was not detected.

As shown previously [11], redox compounds induced rapid decreases in absorbance at the beginning of the reaction, and the velocity slowly declined as the lipid peroxide was consumed, which is the typical pattern for redox inhibitors [Figure 2a]. One example was zileuton, which showed a clear redox pattern with a reduction in absorbance of 0.042. CAY10606 also showed decreases in absorbance, albeit at much weaker signals compared with that of zileuton. Non-redox compounds and DMSO controls showed slight increases in absorbance over time. Caffeic acid displayed no changes in absorbance over time. The other inhibitors showed non-redox patterns of increasing signals [Figure 2, Table 1]. Our findings showed that the absorbance assay yielded results that contradicted with known redox or non-redox patterns of the inhibitors. The most dramatic difference was shown for CDC. It was reported as a redox inhibitor according to the literature [15] and also showed fast consumption of 13(S)-HpODE in our fluorescence assay [Figure 3]. To our surprise, CDC showed perfect non-redox pattern like DMSO and PF4191834. To check whether the difference was originated from different reaction buffer, absorbance assay was also carried out in a buffer used in redox fluorescence assay. Although CDC showed strong redox potential in fluorescence assay, the absorbance change was still increasing pattern even in the same buffer condition [Figure 4-C].

**Table 1:** Comparison of the assay results with the known redox mechanisms of 5-LO inhibitors.

	Known mechanism	Redox fluorescence, EC_50_ (µM)	Redox absorbance, Δabs
Zileuton	Redox	0.45±0.41	−0.025±0.018
PF4191834	Non-redox	>100	0.015±0.005
YS121	Non-redox	77.2±35.1	0.008±0.005
Caffeic acid	Redox	13.9±3.2	0.005±0.003
CDC	Redox	0.13±0.17	0.015±0.005
CAY10606	Redox	12.8±7.8	−0.008±0.005
CAY10649	Non-redox	>100	0.014±0.011
NDGA	Redox	5.5±6.6	0.009±0.007

The results of two redox assays and the known redox mechanisms of tested compounds were compared. The lower EC_50_ value from fluorescence assay signifies the stronger redox activity. When the calculated EC_50_ was over the experimental range, it was indicated as having >100 µM of EC_50_. The change in absorbance (Δ abs) of each inhibitor was calculated by subtracting the absorbance at 180 seconds from the starting absorbance value. The tests were performed in triplicate, and their averages and standard deviations are presented. The known mechanisms are explained in the materials and methods section.

The redox fluorescence assay correctly distinguished the three non-redox inhibitors by having high EC_50_ values. Meanwhile, the redox absorbance assay predicted contradictory results to known mechanism for several redox compounds, such as NDGA and CDC.

The absorbance pattern after substrate depletion was observed by measuring absorbance change after long incubation with redox inhibitor, zileuton [Figure 4-B]. After long incubation (80 minutes), the absorbance at 234 nm was gradually increased just like non-redox control (DMSO) [Figure 4-A].

### Redox fluorescence assay

The accuracy and efficacy of the fluorescence assay were also determined by testing the selected compounds and measuring the amount of remaining 13(S)-HpODE by DCF fluorescence. The fluorescence values of the reaction mixtures ranged from 300 to 6000. The maximum amount of peroxide yielded the highest signal, and the strong redox inhibitor (10 µM) reduced it to 300.

The highest and lowest compound concentrations in the serially diluted set were 100 µM and 1.69 nM, respectively. This range covered the EC_50_ level for all of the tested compounds. The final DMSO amount in the reaction mixture was kept at 1% throughout the experiments. Zileuton was used in each experiment as the positive control. Four wells without any inhibitors represented the “100% peroxide” control wells, and the average fluorescence values in the control wells were used for normalization of fluorescence data. The concentration points were duplicated in each test and the concentration-dependent fluorescence data were fitted with three-parameter logistic regression in Prism using the top and bottom constraints of 100 and 0, respectively.

Various dose-response curves and EC_50_ values were generated in the presence of the tested inhibitors and representative results were shown in the figure 3. Average and standard deviation values were calculated from three independent test of duplicate assay [Table 1]. CDC had the lowest redox potential, with an EC_50_ of 0.13 µM. The EC_50_ of zileuton was about 0.45 µM. The non-redox compounds had EC_50_ values that were higher than 30 µM, suggesting that their redox activities were low or negligible. All redox inhibitors had EC_50_ values that were less than 30 µM.

## Discussion

The redox absorbance assay has been used to qualitatively determine the redox mechanisms of 5-LO inhibitors [10], [11]. Although the method is rapid and easy to handle, it has several shortcomings for practical use. To overcome these limitations, we introduced a fluorescence-based assay in this study.

In the redox absorbance assay, the maximum absorbance change was very small (∼0.05), from 2.338 to 2.296. Although the molar extinction coefficient of 13(S)-HpODE is 23,000 M^−1^•cm^−1^ [19] and a 10 µM solution can give a maximum absorbance change of 0.23, the practical absorbance change was much lower in studies conducted by others and us [10], [11]. One reason for the small absorbance change is that 13(S)-HODE, a product of 13(S)-HpODE consumption, also exhibits absorbance at the same wavelength (234 nm). Therefore, degradation of the peroxide into its corresponding alcohol cannot be reflected by measuring absorbance. In addition, any decreases in absorbance were minimized by the elevated levels exhibited in the presence of DMSO (negative control) [Figure 2]. Reaction components, including the enzyme lysate, buffer components, DMSO, and inhibitor, appeared to contribute to the higher absorbance values at 234 nm. Endpoint measurements could not be made because of the large variation in starting absorbance values. All of these factors suggest that the absorbance assay is inaccurate and can only be used for kinetic purposes. On the contrary, the fluorescence signal of H2DCFDA was not affected by the reaction components and could solely reflect the amount of remaining peroxide. The signal window ranged from as low as 300 to as high as 6000 at the same concentrations of inhibitors that were used in the absorbance assay. The possibility of signal interference seems rare, as it would occur only when the inhibitor fluoresces at the same wavelength as that of DCF. Fortunately, this would be easily recognizable by the abnormal shape of the dose-response curve.

The redox mechanisms of a few inhibitors were ambiguous from the absorbance assay. This was partly due to the increase in negative signal in the absence of redox activity [Figure 2]. The signal increase was also reported by others [11], and may be a result of an unknown reaction in the mixture. Because crude cell lysates were used as the source of 5-LO, molecules that exhibit UV absorbance may have been produced. A set of DMSO controls consistently showed increases in signal in every absorbance assay. In addition, no changes in absorbance were observed with caffeic acid, which is a known redox inhibitor. Overall, many of the observed patterns differed from what is known about the compounds' mechanisms of action [Figure 2, Table 1]. Using the fluorescence assay, we found that the redox activity of caffeic acid was 60% less than that of the stronger redox inhibitors. The dose-response curve of caffeic acid showed its effective concentration (EC_50_) to be 13.9 µM, which was much higher than that of zileuton but lower than that of the three non-redox inhibitors. This suggests that caffeic acid is a weak redox inhibitor.

Because the initial reaction occurs too rapidly for accurate measurements, the absorbance assay may give biased results. Zileuton, a potent redox inhibitor, shows about 50% of the decrease in absorbance in the first 20 seconds, after which the rate slows down [11] [Figure 2]. Several seconds elapsed between the mixing of the solutions and the initiation of absorbance measurements. Much of the decrease in absorbance would have been lost during the initial time interval, especially for strong redox inhibitors. Their patterns may appear to be similar to those of weak redox inhibitors, based on the slow phase of the reaction curve after rapid substrate consumption. We found that zileuton showed increases in absorbance after the peroxide substrate was fully consumed [Figure 4-B]. On the contrary, the fluorescence assay resolved these issues by only measuring the values at the completion of the reaction.

Five of the tested compounds are known to be redox-active. However, according to the absorbance assay, only three appeared to be redox-active. The other two showed non-redox patterns of increasing absorbance. Meanwhile, results from the fluorescence assay showed EC_50_ values that ranged from 130 nM to over 100 µM. The three compounds with the highest EC_50_ values matched those with known non-redox mechanisms [Table 1]. The discrepancies between the absorbance and fluorescence results may be partially explained by the endpoint measurements and high-signal windows. Furthermore, NDGA and CDC showed increases in the absorbance assay, which suggested that they are non-redox compounds. NDGA is a well-known redox compound, and the fluorescence assay revealed that CDC was the strongest redox compound. The low sensitivity of the absorbance assay alone cannot explain the discrepancies between its results and the known mechanisms of action. Different buffer condition was not the reason of disagreement between absorbance and fluorescence assay [Figure 4-C].

The absorbance change is related to the loss of the conjugated system of 13(S)-HpODE. The consumption of 13(S)-HpODE is complex and includes the alkoxide and epoxyallylic radicals [10]. From the unstable radical, several hydroxyl derivatives and cleavage products are produced, some of which can yield absorbance changes at 234 nm. In the given situation, radical scavenging activity may explain the contradictory results of NDGA and CDC. Czapski et al. suggested that strong antioxidants, such as NDGA and baicalein, may work by inhibiting the enzymatic activity of 5-LO and directly scavenging free radicals [20]. Furthermore, they claimed that AA-861 (2-(12-Hydroxydodecane-5,10-diynyl)-3,5,6-trimethyl-p-benzoquinone) and zileuton are weak antioxidants that can serve as specific tools to the 5-LO inhibition study. Czubowicz et al. also suggested that the antioxidant effect should be taken into consideration when evaluating 5-LO inhibitors [21]. It is not rare for inhibitors of same target to have different mechanisms and to have multiple functions [22], [23]. Caffeic acid and its derivatives, such as CDC, have radical scavenging activities [24]. NDGA is a well-known radical scavenger, and its activity was confirmed in studies by Czapski et al. and Czubowicz et al. [20], [21]. Their radical scavenging activities may have caused the intermediate radicals in the redox assay to produce different products. When these resulting products have UV absorbance, the redox absorbance assay can reflect the incorrect results that we have obtained with NDGA and CDC. The fluorescence assay is not affected by product variation because the dye reacts with substrate. By comparing the known mechanisms with the experimental results, we showed that the fluorescence assay is much more reliable in terms of sensitivity and accuracy.

The redox mechanisms of known 5-LO inhibitors were assessed using the absorbance method. We found that the redox absorbance results were easily biased by many factors related to UV absorption, thus leading to inaccurate results. To overcome these limitations, we developed a fluorescence assay, which provides large signal windows and is not easily affected by reaction components, reaction speed, and radical scavenging activities. The assay provided EC_50_ values to evaluate the redox potentials of 5-LO inhibitors. Importantly, it correctly classified the redox mechanisms of eight known inhibitors. In conclusion, the fluorescence redox assay is a better alternative than the conventional, absorbance-based assay for the classification of redox activity for 5-LO inhibitors.
